# Marine Peptides: Structure, Bioactivities, and a New Hope for Therapeutic Application

**DOI:** 10.3390/md19080407

**Published:** 2021-07-23

**Authors:** Tatiana V. Ovchinnikova

**Affiliations:** 1 M.M. Shemyakin & Yu.A. Ovchinnikov Institute of Bioorganic Chemistry, The Russian Academy of Sciences, Miklukho-Maklaya Str. 16/10, 117997 Moscow, Russia; ovch@ibch.ru; Tel.: +7-495-336-44-44; 2 Department of Bioorganic Chemistry, Faculty of Biology, Lomonosov Moscow State University, 119234 Moscow, Russia; 3 Department of Biotechnology, Sechenov First Moscow State Medical University, 119991 Moscow, Russia

Over the last years, plethora of bioactive peptides have been isolated from organisms which live in sea water. Taking in account that more than two-thirds of the global surface area is covered with the oceans, marine species constitute above a half of the total biodiversity. Long-term evolution of marine organisms was advanced in the midst of pathogens, and efficient defense mechanisms were the necessary condition of their survival. Many marine peptides play a key role in host defense as evolutionary ancient components of innate immunity system [1]. They are involved in basic mechanisms of living organisms survival, including growth, defense, reproduction, and homeostasis. Protective peptides were isolated from many marine invertebrates and vertebrates. Their antibacterial, antifungal, antiviral, antitumor, antioxidative, antihypertensive, antiatherosclerotic, anticoagulant, antidiabetic, analgesic, immune-modulating, and neuroprotective properties attract increasing attention of pharmaceutical, cosmeceutical, and nutraceutical industries which focus on the design of innovative antibiotics, anticancer drugs, analgesics, medicines for neurological disorders, etc.

Marine peptides are multifarious in structure and biological properties. The Special Issue «Marine Bioactive Peptides II: Structure, Function, and Therapeutic Potential» was aimed to collect papers on present-day information regarding structural elucidation, functional characterization, and therapeutic potential evaluation of peptides from marine organisms. Getting started with this book, we hope to assemble an interesting edition that would highlight new developments and current trends in marine peptide research.

Cyanobacteria are Gram-negative organisms, also called blue-green algae. They appeared over 3.5 billion years ago and live all over the world including fresh and ocean water, deserts and ice shelves. This evolutionary success is related to their ability to produce a wide variety of secondary metabolites [2]. Lipopeptides laxaphycins have been isolated from several species of cyanobacteria including *Hormothamnion enteromorphoides, Anabaena torulosa, Lyngbya confervoides* and *Anabaena laxa* [3]. In particular, laxaphycins B and B3, and acyclolaxaphycins B and B3 were isolated from the marine cyanobacteria Anabaena torulosa. In this Special Issue, two new acyclic compounds, [des-(Ala4-Hle5)] acyclolaxaphycins B and B3, were purified from the herviborous gastropod (sea hare) Stylocheilus striatus, and structures of both compounds were elucidated [4]. Activities of all the six peptides were determined towards SH-SY5Y human neuroblastoma cells. In this Special Issue, pro-apoptotic properties of cyclic laxaphycins B were indicated. Acyclic laxaphycins affected on autophagy-related protein expression by increasing AMPK phosphorylation and inhibiting mTOR. Gastropod-derived acyclic compounds were shown to undergo a biotransformation (ring opening and amino acid residues deletion) that had not been previously described [4].

Marine fungi represent a valuable source of bioactive peptides. In this Special Issue, the review [5] summarized data on structures and biological activities of 131 peptides isolated from 17 fungal genera including *Acremonium, Ascotricha, Aspergillus, Asteromyces, Ceratodictyon, Clonostachs, Emericella, Exserohilum, Microsporum, Metarrhizium, Penicillium, Scytalidium, Simplicillium, Stachylidium, Talaromyces, Trichoderma,* and *Zygosporium.* 35 marine peptides revealed cytotoxic activities against different cancer cells, 23 ones displayed pronounced antimicrobial and antiviral activities. For example, asperterrestide A exerted the antiviral activity against influenza H1N1 and H3N2 virus strains. Some of the *Aspergillus* peptides revealed anti-inflammatory properties via inhibiting IL-10 expression of the LPS-induced THP-1 cells. Antidiabetic and lipid-lowering activities were also demonstrated some fungal peptides, such as terrelumamides A and B, which improved insulin sensitivity as determined by the utilization of mesenchymal cells of a bone marrow origin obtained from human adopting an adipogenesis model. Psychrophilin G exhibited a pronounced lipid-reducing activity. Simplicilliumtide D displayed an antifouling effect against the larvae of *Bugula neritina*. However, 47% of the isolated peptides did not show the examined biological activities and thus required further in-depth study [5].

Jellyfish are venomous marine invertebrates of the phylum Cnidaria. All members of this ancient phylum are generally toxic (jellyfish, sea anemones, hydra, and corals) [6]. The toxins of these mostly marine animals are of ever-increasing biomedical interest. In this Special Issue, the putative toxins of two species of jellyfish (*Rhopilema esculentum* Kishinouye, 1891, also known as a flame jellyfish, and Amuska jellyfish *Sanderia malayensis* Goette, 1886) were identified in nematocysts [7]. Using nano-flow liquid chromatography tandem mass spectrometry (nLC–MS/MS), in total 3000 proteins were found in the nematocysts in each of the above two jellyfish species. 40 and 41 putative toxins were identified in *R. esculentum* and *S. malayensis*, respectively. All the toxins were further classified into 8 families in accordance with their predicted functions. The most dominant toxins (>60%) were identified as hemostasis-impairing ones and proteases. For the first time ever, the authors studied the proteomes of nematocysts from two jellyfish species [7]. Earlier, this group reported high-quality de novo reference genomes for the above jellyfish, as well as their transcriptomes [8]. Herein, the authors identifed the putative toxins in these two jellyfish species at the protein level. 

Many bioactive peptides were identified in the immune, neuroendocrine, and gut systems of mollusks [9]. The marine peptide Dolastatin 10 (Dol-10) was isolated from the Indian Ocean mollusk *Dolabella auricularia* [10]. The peptide induces apoptosis of lung cancer cells and other tumor cells at nanomolar concentrations. Dol-10 and its derivatives are highly effective in vitro towards L1210 leukemia cells, small cell lung cancer NCI-H69 cells, human prostate cancer DU-145 cells, etc. [11]. Dol-10 has been developed into commercial drugs for treating some specific lymphomas. With the implementation of Dol-10 derivatives and antibody-drug conjugates (ADCs), anticancer activity and tumor targeting were essentially improved, and the systemic toxicity was reduced. Adcetris^®^ has been approved for the treatment of anaplastic large T-cell systemic malignant lymphoma and Hodgkin lymphoma [11]. Thus, Dol-10 is one of the most medically valuable marine peptides discovered up to now. By modifying the chemical structure of Dol-10 and combining the peptide with the application of ADCs technology, new antitumor drug candidates can be developed. In this Special Issue, the authors summarized data on biological activities and chemical structures of Dol-10 derivatives [11]. In their comprehensive review, the authors analyzed synthetic work with Dol-10 in the last 35 years, which provides the input for the development of novel antitumor drugs on the basis of marine peptides. 

Molecular mechanisms of anticancer action of marine peptides are still obscure. Nevertheless, several marine peptides have been applied in preclinical treatment. This Special Issue presents the comprehensive review [12] that highlights mechanisms of anticancer action of linear and cyclic peptides from marine organisms. More than 10,000 bioactive molecules have been isolated from marine organisms [13]. Over recent years, many natural and synthetic peptides were characterized, and special databases were established, including database of anticancer peptides and proteins The CancerPPD [14]. 49 marine-derived bioactive compounds or their derivatives have been applied for clinical trials or approved for the market [15]. 11 marine drugs were approved for the market by European and American drug authorities. Four of them are anticancer drugs: Cytosar-U, Yondelis, Halaven, and Adcetris [12]. The review focus on small anticancer peptides of marine origin and molecular mechanisms of their action with a view to the future development of novel marine antitumor agents. 

The large majority of polychaeta species are marine animals that inhabit all oceans and seas from the Arctic to the Antarctic. Marine polychaeta is an underinvestigated class of invertebrates in the context of discovery of new host defense peptides. Papers included in this Special Issue deal with marine polychaeta, providing good examples of their biological potential. The peptides arenicins and capitellacin, were isolated from *Arenicola marina* [16] and *Capitella teleta* [17], respectively. Earlier, structure-function relationships of arenicins have been extensively investigated [18,19,20,21,22,23,24,25]. Arenicin was shown to modulate the human complement system [26]. Here, the authors reported the property change in arenicin-1 derivative Ar-1-(C/A) structure and its antimicrobial, hemolytic and complement-modulating activities in comparison with those of the natural peptide. Despite the absence of a disulfide bond, the peptide possessed all important functional features, but its hemolytic activity reduced [26]. The use of marine peptides as new complement modulators has several advantages. These molecules are relatively small, and the immune response to them is limited. They do not resemble human peptides, which allows to avoid cross-reactivity and side effects. Besides, this approach promotes antimicrobial effects, taking into account the inhibited complement system [26]. In this Special Issue, a novel BRICHOS-domain related AMP from the marine polychaeta *Capitella teleta*, named capitellacin, was reported [17]. The peptide exhibits high homology with β-hairpin marine peptides tachyplesins and polyphemusins from the horseshoe crabs. The β-hairpin structure of capitellacin was proved by CD and NMR spectroscopy. In aqueous solution the peptide adopts a monomeric right-handed twisted β-hairpin without significant amphipathicity. Moreover, the peptide retains this conformation in membrane environment. Capitellacin displays a pronounced antimicrobial activity in vitro against a wide panel of bacteria including drug-resistant strains. In contrast to other known β-hairpin antimicrobial peptides, capitellacin acts via non-lytic mechanism at concentrations inhibiting bacterial growth. Molecular mechanism of the peptide antimicrobial action does not seem to be related to the inhibition of bacterial translation. A low cytotoxicity towards human cells and high antibacterial cell selectivity as compared to tachyplesin-1 make capitellacin a promising candidate compound for design of a novel anti-infective drug [17]. 

The Spanish mackerel *Scomberomorous niphonius* belongs to the Scombridae family and is distributed in the Western North Pacific, including the East China Sea, the Yellow Sea, and the Bohai Sea of China. Recently, some bioactive ingredients have been prepared from the skins and bones of the Spanish mackerel [27]. Among them, antioxidant peptides (APs) are of particular interest. Eight APs including GPY, GPTGE, PFGPD, GPTGAKG, PYGAKG, GATGPQG, GPFGPM, and YGPM have been earlier isolated from the skin of the Spanish mackerel [28]. In this Special Issue, the authors reported isolation and characterization of APs from the protein hydrolysate of the Spanish mackerel muscle obtained by in vitro gastrointestinal (GI) digestion and evaluated biological properties of the isolated peptides [29]. The proteins of the Spanish mackerel muscle were hydrolyzed with different enzymes and by in vitro GI digestion. Four novel APs, designated as SMP-3, SMP-7, SMP-10, and SMP-11, were isolated and identified as PELDW, WPDHW, FGYDWW, and YLHFW, respectively. All these peptides displayed high radical scavenging activity, lipid peroxidation inhibition ability, and protective effects on plasmid DNA (pBR322DNA) against oxidative damage induced by H2O2 [29]. 

Marine peptides are approved as a food ingredient in Norway. The peptide compounds are manufactured by hydrolysis of the Atlantic cod Gadus morhua fillet [30]. The tablets are produced by Flexipharma AS and based on the marine peptide compound 565952 P from Firmenich Bjørge Biomarin AS. The double-blinded, randomized, controlled trial of the marine protein hydrolysate (MPH) was used to evaluate its effect o on measures of physical function and strength in the elderly. This is one of the first long-term studies of MPH and age-related changes in muscle health. Despite a limited benefit of marine protein hydrolysate on physical function and strength in older adults was delivered, the authors came to the conclusion that a daily intake of 3 g MPH for 6 to 12 months would prevent loss of physical performance, compared with a placebo [30].

In this Special Issue, the review [31] analyzes structural features and biological activities of 253 peptides, mainly from marine food sources. The authors aimed to present the current state-of-art in marine peptides structures, biological activities, and applications, and also to compare them with those isolated from other animal food sources. In summary, the authors concluded that marine organisms have proven to be invaluable sources of peptides with unique structures and diverse bioactivities [31].

The papers included in this Special Issue deal with various marine-derived peptides, providing an informative view of their biomedical potential. The initial call resulted in 32 submissions, and 19 of them were accepted and included in the Special Issue I [32]. Following the success of the first Special Issue, we invited researchers in the field to contribute to the second edition entitled “Marine bioactive peptides II: structure, function, and therapeutic potential”. A range of new marine peptides were isolated and characterized. Most of them displayed broad-spectrum biological activities and therapeutic potential for clinical trials in humans. All the papers presented in this Special Issue II underlined the central role of bioactive peptides in innate immunity of marine organisms as well as their potential for human health care. 

In conclusion, the Guest Editor thanks all the Authors who contributed to this Special Issue, all the Reviewers for evaluating the submitted manuscripts, and the Editorial board of *Marine Drugs,* especially Prof. Dr. Orazio Taglialatela-Scafati, Editor-in-Chief of this journal for his continuous help in turning this Special Issue into reality.
